# Assessing the Relationship between Gestational Glycemic Control and Risk of Preterm Birth in Women with Type 1 Diabetes: A Joint Modeling Approach

**DOI:** 10.1155/2020/3074532

**Published:** 2020-06-24

**Authors:** Resmi Gupta, Jane C. Khoury, Mekibib Altaye, Roman Jandarov, Rhonda D. Szczesniak

**Affiliations:** ^1^Division of Biostatistics and Epidemiology, Cincinnati Children's Hospital, Cincinnati, Ohio, USA; ^2^Department of Biostatistics, University of Cincinnati, Cincinnati, Ohio, USA

## Abstract

**Background:**

Characterizing maternal glucose sampling over the course of the entire pregnancy is an important step toward improvement in prediction of adverse birth outcome, such as preterm birth, for women with type 1 diabetes mellitus (T1DM).

**Objectives:**

To characterize the relationship between the gestational glycemic profile and risk of preterm birth using a joint modeling approach.

**Methods:**

A joint model was developed to simultaneously characterize the relationship between a longitudinal outcome (daily blood glucose sampling) and an event process (preterm birth). A linear mixed effects model using natural cubic splines was fitted to predict the longitudinal submodel. Covariates included mother's age at last menstrual period, age at diabetes onset, body mass index, hypertension, retinopathy, and nephropathy. Various association structures (value, value plus slope, and area under the curve) were examined before selecting the final joint model. We compared the joint modeling approach to the time-dependent Cox model (TDCM).

**Results:**

A total of 16,480 glucose readings over gestation (range: 50-260 days) with 32 women (28%) having preterm birth was included in the study. Mother's age at last menstrual period and age at diabetes onset were statistically significant (beta = 1.29, 95% CI 1.10, 1.72; beta = 0.84, 95% CI 0.62, 0.98) for the longitudinal submodel, reflecting that older women tended to have higher mean blood glucose and those with later diabetes onset tended to have a lower mean blood glucose level. The presence of nephropathy was statistically significant in the event submodel (beta = 2.29, 95% CI 1.05, 4.48). Cumulative association parameterization provided the best joint model fit. The joint model provided better fit compared to the time-dependent Cox model (DIC (JM) = 19,895; DIC (TDCM) = 19,932).

**Conclusion:**

The joint model approach was able to simultaneously characterize the glycemic profile and assess the risk of preterm birth and provided additional insights and a better model fit compared to the time-dependent Cox model.

## 1. Background

Self-monitoring of blood glucose plays a significant role in reduction of perinatal mortality and morbidity in pregnant women with type 1 diabetes mellitus (T1DM) [1, 2]. Tracking blood glucose over the entire pregnancy has provided insight into the effects of poor maternal glucose control on various neonatal outcomes, including preterm birth [3]. Recent studies have shown that the rate of both spontaneous and indicated preterm birth is increased in women with T1DM [4]. The rate of preterm birth ranged from 22% to 45% in women with T1DM, which is greater by four- to eightfold over the frequency of preterm delivery in pregnancies which are not complicated by diabetes [5]. Preterm babies have been shown to have a higher risk of morbidity, mortality, and poor neurological outcome later in life [6]. In addition to the overall increased rate, poor glycemic control was associated with both spontaneous and indicated preterm birth, for mothers with T1DM [7]. Hence, characterizing the glucose profile together with estimating the rate of maternal glucose sampling over the course of the entire pregnancy is an important step along the path to predict the risk of preterm birth outcome and inform T1DM care management practices.

Regulation of glucose is a dynamic process that varies in response to meals, insulin dosage as well as gestational period [8]. In recent years, advancement in technology has improved diabetes self-management practices allowing monitoring of blood glucose on both a programmed and continuous basis. The maternal glucose profile may be considered measurements of an almost continuous-time monitoring process whose outputs are samples of functions or curves. Each curve accounts for blood glucose oscillations that occur throughout the day and perhaps at the same time on different days throughout pregnancy. The longitudinal measurements of the blood glucose level are subject to measurement error as most other self-reported and machine-recorded data, but measurements may be viewed as a noisy version of the true glucose profile.

A common aim in follow-up studies is to characterize the relationship between longitudinal measurements and the event outcomes to gain a better understanding of the risk of an event such as preterm delivery. The time-dependent Cox model [9] is prevalent in the literature for modeling this kind of association. However, the time-dependent Cox model assumes that the covariates are external and are independent of the event mechanism [10, 11]. Moreover, another strong assumption in the time-dependent Cox model is that the time-dependent covariates are measured without error.

In recent years, a joint model has been shown to be an efficient approach to simultaneously model longitudinal data and an observed outcome, often yielding more accurate, informative prediction than traditional models [12, 13]. The joint model approach constructs two submodels, longitudinal and event time, and links the two models by subject-specific random effect terms. The early development of joint models for longitudinal and survival data was primarily motivated from HIV/AIDS clinical trials to model longitudinal CD4 counts and survival data. Classical models such as the linear mixed effects model for longitudinal data and the Cox model for time-to-event data do not consider dependencies between these two different data types (longitudinal and time-to-event data). By simultaneously modeling these two data types into a single model, joint models can infer the dependence and association between the longitudinal and time-to-event outcomes, in order to better assess the effect of a treatment, to quantify uncertainty, and to provide accurate predictions of outcomes. Excellent expositions of joint models have been provided by Wulfsohn and Tsiatis [14], Tsiatis and Davidian [15], De Gruttola and Tu [16], Wang and Taylor [17], Henderson et al. [18], and Brown and Ibrahim [19]. More recent work on joint models includes Rizopoulos et al. [20], Wu et al. [21], Ye et al. [22], Huang et al. [23], and Wu et al. [24].

In addition to being able to characterize the association between longitudinal and time-to-event outcomes using the joint model, dynamic prediction has also gained increasing momentum in clinical research. Because dynamic predictions are individualized and have the ability to appropriately account for possible nonlinearity in each individual longitudinal trajectory, better prospective treatment decisions may become available. In contrast to the linear mixed model, the joint model could provide individualized risk prediction based on the availability of longitudinal information up to that point with the assumption that the individual is event-free until that time.

In the current study, we utilize the aforementioned advantages of the joint model in the shared-parameter framework to characterize the relationship between maternal glucose profile in the longitudinal submodel and preterm birth outcome in the event submodel. Our overall objective is to improve the prediction using the joint model approach while characterizing the relationship between maternal glycemic profiles and preterm birth among women with T1DM. We hypothesize that the joint model would provide better model fit compared to the conventional approach, the time-dependent Cox model. The final joint model will be chosen based on the model fit statistics for different association structures between the maternal glycemic profiles and the risk of preterm birth. Additionally, following Rizopoulos-proposed Monte Carlo approach [25], we will estimate the risk of preterm birth and illustrated how it can be dynamically updated, given that the subject-specific glycemic profiles were available up to the time of prediction.

## 2. Methods

### 2.1. Study Design and Participant Characteristics

The study methods and cohort characteristics have been described in detail elsewhere [26]. Briefly, women with confirmed diagnosis of T1DM who were either pregnant or planning a pregnancy were recruited and enrolled in our Diabetes in Pregnancy study. The women were prospectively followed over gestation as part of a 17-year interdisciplinary program of diabetes in pregnancy between 1978 and 1995 conducted at the University of Cincinnati Medical Center. The study subjects were managed with intensive insulin therapy, involving a split mixed-dose regimen of three to four injections per day using short- and intermediate-acting insulin combined with dietary regulation. After 1981, women were instructed to check blood glucose concentrations 6–8 times a day: fasting, preprandial (before each meal), 90 min postprandial (after each meal), at bedtime, and occasionally at 3 : 00 AM. The current analysis included women who used a reflectance meter through pregnancy and delivered a singleton live fetus beyond 20 weeks of gestation. The glucose measurements recorded between gestational days 50 and 260 were included in the study. Data from profiles corresponding to ne onatal death within 28 days of delivery or presence of a major congenital malformation were excluded from this analysis. Birthweight was measured within the first hour of delivery using an electronic scale (Toledo Scale, Worthington, Ohio). Preterm delivery was defined as delivery prior to 37-week gestation as a result of either spontaneous preterm labor, or preterm premature rupture of membranes, or any other cause classified by a study perinatologist as indicated preterm delivery. Glycosylated hemoglobin A1 concentration was measured every 4 weeks throughout pregnancy and prior to delivery using Isolab column chromatography. Mother's age at last menstrual period, age at diabetes onset, body mass index, white classification (widely used to assess maternal and fetal risk complicated by diabetes), chronic hypertension, retinopathy, and nephropathy were included as predictors in the initial longitudinal (glucose trajectory) and event (preterm birth) models. Bivariate analysis indicated that mother's age, age at diabetes onset, and the presence of nephropathy were statistically significant in both longitudinal and event models and will be included in the joint model and time-dependent Cox modeling phase. Glucose recordings were available for all patients on the day the event (birth) occurred.

### 2.2. Statistical Methods

Each joint model consists of two linked submodels, a mixed effects model to fit longitudinal blood glucose monitoring and a survival model to fit preterm birth data. The longitudinal outcome, glucose recordings (log-transformed scale) from the *i*^th^ woman and *j*^th^ pregnancy observed at gestation time *t*_ij_ (*i* = 1 ⋯ .*n*, *j* = 1 ⋯ *n*_*ij*_) can be expressed as

(1) $$\begin{array}{c} \text{Glucose }{\text{recording}}_{ij}=X_{ij}^{T}\left(\theta \right)+f\left(t_{ij}\right)+Z_{ij}^{T}b_{i}+\varepsilon_{ij}, \end{array}$$

where parametric effects are represented for traditional covariates *X*_*ij*_ and *p* × 1 parameter vector *θ*; *f*(.) is a nonparametric smooth function evaluated at time *t*_*ij*_; *Z*_*ij*_, and *b*_*i*_ corresponding to the design matrix and subject-specific *q*_*i*_ × 1 parameter vectors for random effects; *ε*_*ij*_ represents the measurement error corresponding to the observation at time *t*_*ij*_. The mean response function *f*(*t*_*ij*_) in equation (1) below can be estimated using the natural cubic spline which can be expressed as: *f*(*t*_*ij*_) = ∑_*r*=0_^22^*B*_*r*_*λ*_*r*_(*t*_*ij*_) where *B*_*r*_ are the parameter coefficients of the expansion with basis functions *λ*_*r*_(*t*_*ij*_). In order to capture the nonlinear subject specific fluctuations in glucose recordings, we included the natural cubic spline that expands the time effects into a B-spline basis matrix. Based on the Bayesian information criteria [27], we selected a total of 22 knots, which was necessary to estimate the individual dynamic prediction for risk of preterm birth (discussed in the next section). Including the fixed-effect covariates, maternal age at last menstrual period, age at diabetes onset, and spline functions, the above equation can be written as

(2) $$\begin{array}{c} \text{Glucose }{\text{recording}}_{ij}=m_{i}\left(t\right)+\varepsilon_{i}\left(t\right)=\left(\beta_{0}+b_{i0}\right)+\left(\beta_{1}+b_{i1}\right)B_{r}\left({\left(\text{gestational day}\right)}_{ij}+\lambda_{1}\right)+\left(\beta_{2}+b_{i2}\right)B_{r}\left({\left(\text{gestational day}\right)}_{ij}+\lambda_{2}\right)+\cdots \cdots +\left(\beta_{22}+b_{i22}\right)B_{r}\left({\left(\text{gestational day}\right)}_{ij}+\lambda_{22}\right)+\beta_{23}{\left(\text{age}\right)}_{i}+\beta_{24}{\left(\text{age at diabetes onset}\right)}_{i}+\varepsilon_{ij}. \end{array}$$

In equation (1), *B*_*r*_ represent the fixed effects part of the spline coefficient, *r* = 0, 1, ⋯22. We assumed that the random spline coefficients are mutually independent and distributed as *b*_*ik*_ ~ *N*_23_(0, *σ*_*ik*_), *k* = 0, 1, ⋯22, and measurement error *ε*_*ij*_ ~ *N*(0, *σ*_*ε*_^2^).

We linked the event model, probability of preterm birth to the glucose measurements through the random effects. Let *h*_*i*_ denote the event endpoint, which is the binary indicator of preterm birth. The probability of preterm birth is linked to the longitudinal process of glucose measurements as

(3) $$\begin{array}{c} h_{i}\left[t\right]={lt}^{l-1}\exp\left\{\Omega_{i}^{T}\left(\phi \right)+\alpha m_{i}\left(t\right)\right\}, \end{array}$$

where *Ω*_*i*_^*T*^ denotes the vector of fixed effects covariates, such as the presence of nephropathy at baseline; *m*_*i*_(*t*) is the true values of the underlying glucose monitoring processes, with *α* being the parameter characterizing the association between longitudinal glucose recording process and the PB outcome. Because of model complexity we specified the Weibull distributional form for the baseline hazard function. Equation (3) monotonically increases with time if l > 1, decreases if l < 1, and the exponential hazard remains constant if l = 1.

In equation (3), the risk of preterm birth depends on the current value of the blood glucose level. However, since for each patient the blood glucose follows a trajectory in time, it is also reasonable to consider a joint model in which the risk depends on both the current true value of the blood glucose trajectory and the slope of the true blood glucose trajectory at that time. The event model then becomes

(4) $$\begin{array}{c} h_{i}\left[t\right]={lt}^{l-1}\exp\left\{\Omega_{i}^{T}\left(\phi \right)+\alpha_{1}m_{i}\left(t\right)+\alpha_{2}m_{i}\left(t^{\prime }\right)\right\}, \end{array}$$

where *m*_*i*_(*t*′) = (*d*/*dt*)(*m*_*i*_(*t*)). Additionally, we fitted the association structure to allow the whole history of the blood glucose trajectory to be associated with the risk of the preterm birth. Then, the event model takes the form

(5) $$\begin{array}{c} h_{i}\left[t\right]={lt}^{l-1}\exp\left\{\Omega_{i}^{T}\left(\phi \right)+\alpha \int_{0}^{t}m_{i}\left(s\right)ds\right\}. \end{array}$$

For any particular time point, *α* in (4) measures the strength of association between the risk of PB at time *t* and the area under the longitudinal trajectory of the blood glucose level up to the same *t*. We tested all three association structures (value, slope, and area under the curve) and selected the best model based on the deviance information criteria (DIC) [28]. The deviance information criteria measure balances, the fit of a model to the data with its complexity. A smaller value of DIC indicates the preferred model.

We compared the joint model with the time-dependent Cox model where we fitted the preterm birth event model with a Cox model that included glucose measurements as a regular time dependent covariate (ignoring measurement error), i.e.,

(6) $$\begin{array}{c} h_{i}\left[t\right]={lt}^{l-1}\exp\left\{\gamma_{1}{\left(\text{glucose}\right)}_{ij}+\gamma_{2}{\left(\text{gestational day}\right)}_{ij}+\gamma_{3}{\left(\text{glucose}\right)}_{ij}*{\left(\text{gestational day}\right)}_{ij}+\gamma_{4}{\left(\text{age}\right)}_{i}+\gamma_{5}{\left(\text{age at diabetes onset}\right)}_{i}+\gamma_{6}{\left(\text{presence of nephropathy}\right)}_{i}\right\}. \end{array}$$

Model comparison between the time-dependent Cox model and the joint model was conducted by DIC. All data analyses were conducted using SAS v9.4.4 (Cary, NC) and JMbayes package in RStudio (2015), where the time-dependent Cox model was estimated using a frequentist procedure, and the joint models were implemented using the approach available in JMbayes. We used readily available software to estimate the model parameters. The default prior distribution was used for JMbayes to estimate all joint model parameters. We preferred to use JMbayes instead of the JM software package, since the former is actively maintained and provides straightforward implementation of dynamic prediction [12]. Estimates from each model are reported with 95% confidence or credible intervals (denoted as 95% CI), depending upon whether the model was implemented with the frequentist or Bayesian approach.

### 2.3. Individual Dynamic Prediction

In this paper, we used Rizopoulos-proposed approach [25] to dynamically update the risk of preterm birth based on the updated glucose trajectory for an individual subject. A key feature of these dynamic prediction frameworks is that the predictive measures for the risk of preterm birth can be dynamically updated as additional longitudinal measurements of the blood glucose level become available for the target subjects, providing instantaneous risk assessment.

## 3. Results

Descriptive statistics are presented in Table 1. A total of 114 women with T1DM with 16,480 glucose readings were included in this analysis. The median (IQR) number of glucose recordings was 157 (6-211); 32 (28%) of the women had preterm delivery. Median age of entry was 27 (22–31) years; median age of diabetes onset was 12 (8–17) years; median baseline BMI of the cohort was 23 (21–25) kg/m^2^. A total of 32 (28%) were diagnosed with preeclampsia, 14 (12%) with chronic hypertension, 40 (35%) with white classification (B/C vs ≥D), 22(19%) with nephropathy, and 16 (14%) with retinopathy.

**Figure 1:**
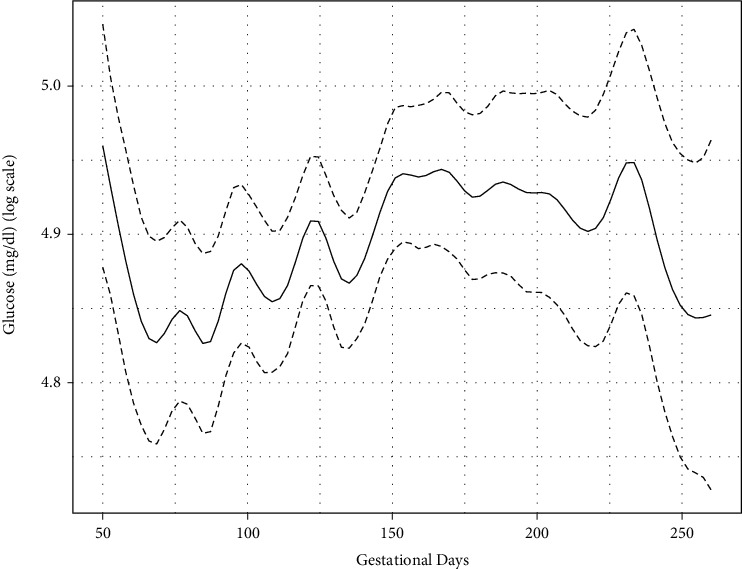
Fitted glucose model (on log scale) with natural spline over 50-260 gestational days.

In order to allow for flexibility in the nonlinear blood glucose profiles, we included a natural cubic splines in both the fixed and random effects part of the mixed effects model. A range of knots was used to fit the longitudinal glucose profiles. Based on Bayesian information criteria, a total of 22 knots was selected to fit individual profiles.  Figure 1 corresponds to the overall model fit for the glycemic profiles and 95% CI for the period of 50-260 gestational days. Mother's age at last menstrual period (*β*_age_ = 1.29, 95% CI 1.10, 1.72) and age at diabetes onset (*β*_ageatdiabetesonset_ = 0.84, 95% CI 0.62, 0.98) were statistically significant for the longitudinal submodel, reflecting that older women tended to have a higher mean blood glucose level and those with later diabetes onset tended to have a lower mean blood glucose level (Table 2). The presence of nephropathy was statistically significant for the event submodel, reflecting that the probability of preterm birth significantly increases with the presence of nephropathy among women with T1DM (*RR*_neph_ = 2.29, 95% CI 1.05, 4.48). Based on deviance information criteria, the joint model provided better model fit in comparison to the time-dependent Cox model (DIC_JM_ = 19895; DIC_TDCM_ = 19932). Unlike the joint model, mother's age and age at diabetes onset were not significant in the time-dependent Cox model. The presence of nephropathy was statistically significant in the time-dependent Cox model (RR_neph_ = 2.21, 95% CI 1.01, 3.32).

Based on deviance information criteria, the association link with cumulative glucose monitoring provided the best fit (DIC_*α*_Value__ = 19, 990; DIC_*α*_Value plus slope__ = 19, 905; DIC_*α*_AUC__ = 19, 895) compared to the value or rate of the glucose level at time *t* (Table 3). The cumulative association (area under the curve) indicated that the cumulative effects of the glucose monitoring outcome up to time point *t* had the strongest association (*α*_AUC_ = −0.01, *p* < 0.01) between the risk of preterm birth and maternal glycemic profile.

**Figure 2:**
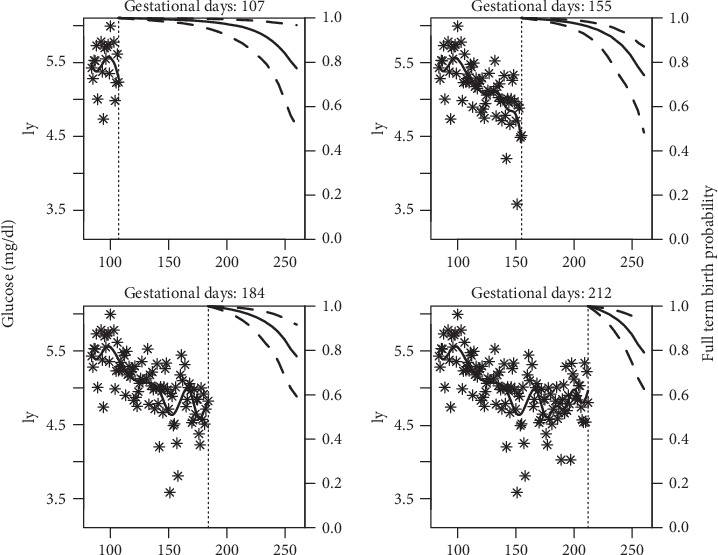
Individual dynamic prediction of risk for preterm birth with updating blood glucose readings (on log scale).

The assumption for dynamic prediction is that the woman was event-free (i.e., did not give birth) up to the time point of the last glucose reading available. Using the Markov chain Monte Carlo algorithm, the estimates of the risk of preterm birth were computed for a new subject knowing her glucose readings up to a given gestation day.  Figure 2 depicts the change in the risk of preterm birth of a woman with an increasing number of glucose readings over time. For glucose readings up to 107 gestational days, the woman had roughly a 25% risk of having premature delivery. The risk decreases by 5% with her available glucose readings out to 212 gestational days in pregnancy.

## 4. Discussion

In this study, we demonstrated that the characterizing and estimating rate of maternal glucose sampling profile over the course of the entire pregnancy is an important task to predict preterm birth outcome. Recent studies have shown that the rate of both spontaneous and indicated preterm birth is increased in women with T1DM [29, 30]. Preterm babies go on to have a higher risk of increased morbidity, mortality, and poor neurological outcome [31]. In mothers with T1DM, poor glycemic control was associated with both spontaneous and indicated preterm birth [32]. As we have shown in our previously published work [8], nonlinear curvature of blood glucose monitoring data makes it analytically challenging. In recent years, the joint model has been shown to be an efficient approach to simultaneously model longitudinal data and an observed outcome, often yielding more accurate, informative prediction than traditional models [12]. In this paper, we describe a joint model approach aimed at analyzing long sequences of longitudinal and time-to-event data and used it to simultaneously characterize the glycemic profile for women with T1DM and assess risk of preterm birth. On the population level, we identified demographic characteristics associated with longitudinal and event outcomes. When comparing the joint model approach to the commonly used time-dependent Cox model, we found that the former was a better fit based on the model fit statistics and characterized association between the risk of preterm birth and glucose measurements over the entire pregnancy period to provide additional insights compared to the time-dependent Cox model. While maternal age at last menstrual period and age at diabetes onset were not significant in the time-dependent Cox model, it was significant in the joint model providing additional information regarding the relationship between maternal glycemic profile over the course of entire pregnancy and mother's age at last menstrual period and age at diabetes onset. We found that older women tended to have higher mean blood glucose, and those with later diabetes onset tended to have lower mean blood glucose. The presence of nephropathy was statistically significant in both the joint model and the time-dependent Cox model.

We tested various association structures to share the parameters between the longitudinal and event submodel within the joint modeling framework. Based on deviance information criteria, the association structure based on area under the curve of the glycemic profile provided the best fit for the model in comparison to the value and value and rate association structures. An alternative criterion for Bayesian joint models is the logarithm of the pseudo likelihood (LPML) [33], which may be computed separately for the submodels using a Monte Carlo approach; however, readily available software does not include either version of the LPML. BIC may be used to rank joint models but, as the authors of the LPML approach describe, it may not be considered in submodel-specific assessments without proper decomposition. The cumulative association structure, as defined by the area under the curve, is clinically more appealing as it includes the cumulative effects of the glucose monitoring outcome up to a certain time point to determine the risk of preterm birth of a subject. Further, our approach, which includes a linear mixed effects submodel, accounts for missing glucose data. This is not possible in summary calculations, which are prone to measurement error [34].

A novel use of joint models, leveraged in this study, is to obtain dynamic personalized prediction of future longitudinal outcome trajectories and risks of an event at any time, given the subject-specific outcome profiles up to the time of prediction. Rizopoulos proposed a Monte Carlo approach [25] to estimate risk of a target event and illustrated how it can be dynamically updated. We followed this approach to show how dynamic prediction for the risk of preterm birth can be obtained as a function of maternal glycemic profile over the course of an entire pregnancy for a woman in this T1DM cohort. Based on the fitted joint model, we derived the prediction for a new subject from the same population that provided a set of longitudinal glucose measurements up to a specified time. As an example, we considered one maternal glycemic profile and, based on a Monte Carlo procedure, showed the mean and median estimates over the Monte Carlo samples along with the 95% pointwise credible intervals. We calculated the conditional full-term birth (i.e., event-free) probabilities for an individual patient, starting from her first glucose measurements and adding glucose at each time point as an extra measurement.

There are some limitations to the current study. We conducted variable selection based on a one-at-a time model selection process. Although this approach is suitable for prediction modeling, it cannot be used to infer causality; therefore, association estimates from this study must be viewed cautiously [35]. With respect to missing data in maternal glycemic profiles, our approach assumes that data are missing at random [36] in the sense that missingness can be explained by observed information. By using the existing R package, JMbayes, we modeled observed birth outcome as a survival process, with no censoring in the data. Our further research is aimed at rectifying this problem by including the submodel as a binary process. The joint model [12, 13, 37, 38] approach proposed in this study may be used to model continuous glucose monitoring data, thus incorporating the cutting-edge technology in diabetes care. Moreover, future work should aim to separately model the irregular glucose sampling pattern associated with the blood glucose level for women with T1DM to predict the risk of preterm birth. Another interesting future work area will be to look into the effects of glycohemoglobin along with glucose measurements to predict the risk of preterm birth using the joint modeling technique.

## 5. Conclusions

We have developed a joint model approach that, once applied to motivating data, provided more realistic estimates of the maternal glycemic profile over the entire pregnancy and enabled individualized assessment of preterm birth risk in women with T1DM. Through a Bayesian Markov chain Monte Carlo algorithm implemented in open-source software, this model could be used to aid clinicians in estimating the risk of preterm delivery sooner than later based on the glucose trajectory and other baseline covariates, potentially allowing for intervention strategies to be applied. We demonstrated that overall model fit improved with our joint model, compared to the conventional time-dependent Cox model. Some important covariates, mother's age at LMP and age of diabetes onset, were significant in the joint model unlike using the conventional approach, the time-dependent Cox model. Accurately characterizing the association between blood glucose level and preterm birth may serve as a prognostic aid for clinical decision making allowing for research focused on personalized, preemptive treatment and monitoring of pregnancies impacted by T1DM.

## Figures and Tables

**Table 1:** Patient characteristics (*n* = 114; 16,480 glucose readings over gestation 50-260 days).

	Median (IQR)	*f* (%)
Mother's age at LMP	27 (22-31)	
Age at diabetes onset	12 (8-17)	
Baseline BMI	23 (21-25)	
Number of glucose recordings	157 (6-211)	
PB		32 (28)
Preeclampsia		32 (28)
White classification		
B, C		40 (35)
≥D		74 (65)
Presence of nephropathy		22 (19)
Presence of hypertension		14 (12)
Presence of retinopathy		16 (14)

IQR: Interquartile range; *f*: Frequency; BMI: body mass index (kg/m^2^).

**Table 2:** Parameter estimates and model fit statistics from the joint model and the time-dependent Cox model.

	Time-dependent Cox model			Joint model		
				LSM	ESM	
	EST (CI)	OR (CI)	M-Val	EST (CI)	OR (CI)	M-Val
Mother's age at LMP	1.02 (0.95, 1.10)			1.29 (1.10, 1.72)^∗^		
Age at diabetes onset	0.97 (0.92, 1.03)			0.84 (0.62, 0.98)^∗^		
Presence of nephropathy		2.21 (1.01, 3.32)^∗^			2.29 (1.05, 4.48)^∗^	
Association						-0.01^∗^
DIC			19,932			19,895
-2Log-likelihood			18,464			19,434

∗*p* < 0.05; CI refers to confidence and credible interval for the time-dependent Cox model and the joint model, respectively (see Methods); LSM: longitudinal submodel; ESM: event submodel; EST: estimate; OR: odds ratio, M-Val: model value.

**Table 3:** Value, value plus slope, and area under the curve association links and model fit statistics for the joint models.

Association link	DIC	pD
Value	19990	2182
Value and slope	19905	2143
Area under the curve	19895	2130

DIC: deviance information criteria; pD: effective number of parameters. Lower values indicate better model fit.

## Data Availability

The data used in the present study to support the findings of this study are available from the corresponding author upon request.
